# Dietary fibre intakes and reduction in functional constipation rates among Canadian adults: a cost-of-illness analysis

**DOI:** 10.3402/fnr.v59.28646

**Published:** 2015-12-11

**Authors:** Mohammad M. H. Abdullah, Collin L. Gyles, Christopher P. F. Marinangeli, Jared G. Carlberg, Peter J. H. Jones

**Affiliations:** 1 Department of Human Nutritional Sciences, University of Manitoba, Winnipeg, MB, Canada; 2 Richardson Centre for Functional Foods and Nutraceuticals (RCFFN), University of Manitoba, Winnipeg, MB, Canada; 3 Department of Agribusiness & Agricultural Economics, University of Manitoba, Winnipeg, Manitoba; 4 Pulse Canada, Winnipeg, Manitoba, Canada

**Keywords:** dietary fibre, constipation, healthcare, cost, savings, nutrition economics

## Abstract

**Background:**

Evidence-based research highlights beneficial impacts of dietary fibre on several aspects of the gut pathophysiology that are accompanied by a considerable financial burden in healthcare services. Recommended intakes of dietary fibre may thus associate with financial benefits at a population level.

**Objective:**

We sought to systematically assess the potential annual savings in healthcare costs that would follow the reduction in rates of functional constipation and irregularity with increased dietary fibre intakes among Canadian adults.

**Design:**

A cost-of-illness analysis was developed on the basis of current and recommended levels of fibre intake in Canada, constipation reduction per 1 g fibre intake, proportion of adults who are likely to consume fibre-rich diets, and population expected to respond to fibre intake. Sensitivity analyses covering a range of assumptions were further implemented within the economic simulation.

**Results:**

Our literature searches assumed a 1.8% reduction in constipation rates with each 1 g/day increase in fibre intake. With intakes corresponding to the Institute of Medicine's adequate levels of 38 g/day for men and 25 g/day for women, among 5 and 100% of the adult populations, anywhere between CAD$1.5 and CAD$31.9 million could be saved on constipation-related healthcare costs annually. Each 1 g/day increase in dietary fibre was estimated to result in total annual healthcare cost savings that ranged between CAD$0.1 and CAD$2.5 million.

**Conclusions:**

The present research suggests an economic value of increasing dietary fibre intake beyond its well-known health benefits. Healthy-eating behaviours consistent with the recommended intakes of dietary fibre by the general public should hence be advocated as a practical approach for reducing costs associated with the management of constipation in Canada.

Dietary fibre is a term that describes the non-digestible carbohydrates from plant sources (1), and is a key component of a healthy diet (2). Considerable evidence highlights beneficial impacts of dietary fibre intakes on a multitude of public health priorities that affect large proportions of populations worldwide, and are accompanied by escalating expenditures in healthcare services (3). Similar to other Western countries, levels of dietary fibre intake in Canada are below the Institute of Medicine's (IOM's) adequate intake of 14 g/1,000 kcal for all age groups, or 21–38 g/day for adults (4). A closer adherence to the targeted levels of fibre intake has the potential to move some of the economic burden of disease from the publicly funded healthcare system and into the grocery store, thus ‘demedicalising’ some of the costs associated with the chronic diseases of relevance.

Chronic idiopathic constipation, or functional constipation, is defined under the Rome III criteria (5) as the diagnosis of two or more of symptoms, that include straining on defecation, hard stools, incomplete evacuation, fewer than 3 bowel movements in a week, or rare loose stools without the use of laxatives, at least some of the time within 3 months. Constipation is a common health problem (6–8) that affects up to 27% of the population of Western countries (9). In Canada, one in four Canadians experiences symptoms of constipation, and approximately 1 million physician visits happen as a result of this each year, resulting in total annual healthcare costs of nearly CAD$7,500 on a per constipated patient (10). Furthermore, Canadians spend approximately CAD$100 million annually on laxatives (10). Increasing dietary fibre intake is commonly recommended as a first tactic in the management of constipation, with 25 g/day considered effective (11). Mechanistically, the water-retaining capacity of fibres is thought to stimulate the gastrointestinal motility by increasing faecal volume or bulk, gut microbiome growth, and the concentration of products from bacterial metabolism. Accordingly, fibre promotes colonic propulsion, reduces transit time, and facilitates defecation (12). As such, the favourable effects of dietary fibre intake on constipation are widely accepted and evident by large cohorts (13, 14) and smaller dietary interventions (15, 16).

Articles on nutrition economic approaches (17, 18) support the notion that nutrition can play a critical role in improving the efficiency and sustainability of healthcare systems. In this regard, Schmier et al. (19, 20) have recently demonstrated significant constipation-related cost savings with increased dietary fibre consumption in the United States and the European Union. However, data on the economic benefits of greater habitual or recommended fibre intakes remain limited, particularly in Canada. In light of the current expansion in popularity, production, and marketing of fibre-rich food products, alongside the well-established health benefits of fibre intakes, the objective of this study was to evaluate the economic value of increased intakes of dietary fibre that could result in reductions from healthcare costs associated with chronic constipation and irregularity in Canada.

## Methods

### Study design

A cost-of-illness analysis entailed three steps of estimation to evaluate the constipation-related healthcare dollar savings associated with increased fibre intakes among Canadian adults. First, a ‘success rate’ was determined to reflect the proportion of individuals who are likely to purchase and consume fibre-rich foods. Using current data and recent consumer trends, fibre intake scenarios were created. Second, the available nutrition literature was reviewed to assess increased dietary fibre intake in relation to beneficial effects on constipation incidence. Finally, with the use of most recent national monetary estimates, the potential savings in healthcare costs associated with the estimated reductions in incidence of constipation and irregularity were computed. As previously (21), a sensitivity analysis was conducted on all parameters described above ranging from a very pessimistic rate (worst-case scenario) to a universal fortification rate (best-case scenario), regarding the fibre intake adoption rate, impact of fibre on constipation, and the healthcare cost flexibility. Further details around the cost-of-illness analysis are discussed below.

In this economic model, three sets of analyses were completed. First, an analysis was conducted to reflect the cost reductions in constipation-related healthcare services when the current actual intakes of dietary fibre for Canadian men (19.1 g/day) and women (15.6 g/day), as reported by Belanger et al. (22), were increased to the IOM's adequate intake cut-offs of 38 and 25 g/day for men and women, respectively (4). These are the cut-off values that policy makers, dietitians, and other healthcare providers in Canada and the United States typically use as guidelines. The second analysis examined the healthcare cost savings per gram increases in fibre intake. For the third analysis, the total dollar savings at incremental levels of 20, 25, 30, and 35 g fibre/day for men and women alike were studied. These incremental thresholds correspond to moderate increases in fibre consumption and represent judicious dietary goals for a range of consumers. This economic model was applied to Canadian adults, who were defined as men and women ≥18 years of age. Population data were attained from Statistics Canada 2014 estimates (23). Table 1 summarises the input parameters of the model.

**Table 1:** Summary of the input parameters for the cost savings assessment model utilised

Parameter	Men	Women	Source
Current fibre intake, g/day	19.1	15.6	Belanger et al. (22)
Target fibre intake, g/day			
	38	25	IOM (4)
	20		Assumption for valuation
	25		of incremental intakes
	30		
	35		
	20.1	16.6	Assumption for valuation of per gram intake
Constipation reduction per 1 g fibre intake, %	1.8	1.8	Dukas et al. (13)
Population expected to respond to fibre intake, %	85	85	Voderholzer et al. (40)

IOM, Institute of Medicine.

### Step 1 of the cost-of-illness analysis: assessment of the ‘success rate’

The public–economic benefit of increased dietary fibre intake is dependent on decisions made by the individual consumer in the marketplace. Accordingly, any model that attempts to measure the potential public health benefit of a lifestyle intervention should begin with insights around consumer behaviour. In the case of this study, the success rate of the dietary behaviour refers to the proportion of the population that ‘chooses’ to consume a healthy level of dietary fibre. A limited amount of research is available with respect to consumer behaviour as it relates to the intake of dietary fibre. Mialon et al. (24) studied the effect of fibre information on consumer responses to breads and English muffins in Australia, and found that the likelihood of consumption of these foods was enhanced when products were labelled as high in fibre. Dean et al. (25) examined perceptions around healthy grain foods in Finland, the UK, Germany, and Italy. When the benefits of grain-based foods were communicated as health claims, consumers’ positive perceptions of grain foods were increased. Baixauli et al. (26) reported higher scores on acceptability and intent-to-purchase for muffins, when information on dietary fibre was provided to consumers on labels. Tudoran et al. (27) explored individual health-value attitudes towards a fibre-enhanced fish product in Spain and demonstrated that consumers scored higher when they were aware of the fibre and health information of the product. Finally, Ginon et al. (28) assessed how fibre-health information impacted the willingness to pay for baguettes in France and observed that reservation prices increased when a ‘source of fibre’ label was presented. However, no such effect was observed when information regarding the potential health benefits of fibre was given. Given that the present analysis is focused on the population in Canada, data corresponding to Canadians’ perceptions of fibre in foods would be beneficial for the assumptions that comprise this analysis. While limited knowledge is available, recent findings from Wong et al. (29) showed that, across all types of health claims, Canadians responded positively to foods that communicated the presence of oat fibre. Fibre is thus generally viewed as a favourable component of food and, for the most part, consumers are receptive to fibre-based health claims.

Given that actual behaviours for long-term intakes of dietary fibre have not been measured, as part of the present sensitivity analysis, assumptions around the proportion of the Canadian population that would adopt adequate levels of fibre intake were made. Based on findings from previous research (24–29), universal, optimistic, pessimistic, and very pessimistic success rate scenarios were modelled. The universal fortification scenario assumes a 100% success rate, i.e. 100% of Canadians would increase their consumption of dietary fibre, and represents a dramatic shift in the dietary habits. While this success rate is not realistically achievable in the short-term, it represents the maximum potential of economic savings with increased fibre intake over the very long-term. The optimistic scenario assumes that 50% of Canadians would increase their consumption of dietary fibre, and denotes a medium- to short-term pragmatic estimate of the potential savings possible through increased dietary fibre intakes. The pessimistic and very pessimistic scenarios are set at 15 and 5% success rates, respectively, and assume that 15 and 5% of Canadians would increase their dietary fibre intakes. While the former represents a less positive (yet still practical) short- to medium-term estimate of economic savings, the latter is included to determine the impact on the cost estimates when assumptions are more pessimistic than normal following increased fibre intake.

### Step 2 of the cost-of-illness analysis: estimation of constipation reduction due to increased dietary fibre intake

Similar to Step 1 of the cost-of-illness analysis outlined above, possible rates of reduction in incidence of functional constipation following higher intakes of dietary fibre were established based on data in the literature. Searches using PubMed were conducted to identify epidemiological and intervention studies that reported associations between dietary fibre and constipation. Meta-analyses were also evaluated. To better predict disease reduction estimates for constipation and account for the rapid evolution of food technologies, globalisation of the food supply, and changing dietary habits, the literature search focused on the English-language studies that were published after the year 2000 principally in developed countries.

Observational research has consistently established beneficial impacts of enhanced dietary fibre intake on rates of constipation. Two large epidemiological studies stand out. In the first, involving a subset of women (*n*=62,036) from the Nurses’ Health Study, Dukas et al. (13) reported a 36% lower prevalence of constipation (PR=0.64, 95% CI 0.57–0.73) among women with the highest quintile of total dietary fibre intake (median intake 20 g/day) versus those in the lowest quintile (median intake 7 g/day). This translated into a 1.8% reduction in constipation prevalence per 1 g/day increase in fibre intake. The second study, a cross-sectional analysis of data from the EPIC–Oxford study, revealed that among individuals with the highest levels of fibre intake (>20.9 g/day), men and women had a 100% (OR=2.00, 95% CI 1.38–2.90) and 43% (OR=1.43, 95% CI 1.24–1.64) increases in odds of having daily bowel movements per week, respectively (14). This translates into approximately 3.4% reduction in constipation per 1 g/day increase in fibre intake.

Dietary fibres are usually classified as soluble or insoluble, depending on their solubility in water. Soluble (or viscous) fibres, for which the major food sources are fruits, oats, barley, and legumes, include gums, pectins, and some hemicelluloses (i.e. beta-glucans). Soluble fibres are commonly believed to act at a metabolic level leading to positive effects on established biomarkers of health, such as delaying glucose absorption and lowering blood cholesterol concentrations (30). Insoluble fibres, on the other hand, are better known for their beneficial impacts on the health of the digestive system by promoting bowel movements (regularity) and accelerating the transit time through the colon. Insoluble fibres include cellulose, many hemicelluloses, and lignins, and are mainly found in wheat bran, whole-grain breads and cereals, and vegetables (30).

A number of dietary intervention studies examined the effects of soluble and insoluble fibre intakes, from various dietary sources, on chronic constipation (31–37), and generally demonstrated favourable effects of dietary fibre on constipation, with reduction rates ranging between 13% (33) and 27% (32) overall, or 0.4% (31) and 4.0% (36) per 1 g intake per day. These findings are complemented by meta-analytic data from Yang et al. (38) where a 19% increase in stool frequency (OR=1.19, 95% CI 0.58–1.80) was reported with increased intakes of dietary fibre, compared to control diets. Whether the effect size remains consistent across the entire range of fibre intakes is at this stage uncertain. For the purpose of this analysis, it was assumed that for each gram increase in dietary fibre, prevalence of constipation is conservatively decreased by 1.8% (13) (Table 1).

### Step 3 of the cost-of-illness analysis: estimation of the potential savings in constipation-associated healthcare costs

The economic cost of disease in Canada is generally broken down into direct and indirect categories. Direct costs are those incurred by the healthcare system with the goal of improving and/or preventing a patient's health status from deteriorating; these usually include hospital care, drug, physician visit, and, sometimes, other miscellaneous costs. Indirect costs, by comparison, are commonly known as those incurred by the loss of productivity arising from mortality and morbidity. A reduction in constipation rates will result in fewer healthcare and related resources being used to treat this highly prevalent condition.

In a 2001 survey, an estimated 27% of the Canadians reported constipation in the previous 3 months, with 34% of afflicted individuals claiming to have sought medical help for the condition (6). The Canadian Institute for Health Information (CIHI) keeps detailed information on hospitalisation and provincially insured drug costs used to treat constipation; these are high-quality data based on provincial billing records. Since the information is not provided for all provinces, the estimate used in this report was adjusted based on Statistics Canada population data to reflect a national average. Additionally, the prevalence estimates developed by Pare et al. (6) were used as a basis for family physician visits with costs based on CIHI data. The potential for indirect costs due to constipation does exist. Indeed, research in the United States reports that 12% of survey respondents suffering from constipation indicated reduced productivity at work or school (39). However, the productivity losses and associated costs for Canada are not well-understood and were thus excluded from the present research.

Table 2 provides the estimated annual direct healthcare costs associated with constipation in Canada. The largest cost was for drugs, estimated at CAD$80.0 million. Physician care costs ranked second at CAD$73.1 million, followed by hospitalisation and serious hospitalisation at CAD$39.4 and CAD$21.2 million, respectively. Constipation-related healthcare cost was assumed to demonstrate a linear reduction with reduced prevalence of constipation within the Canadian population. Similar to the recent analysis by Schmier et al. (19), it was further assumed that only 85% of the population would respond to fibre (40).

**Table 2:** Estimated direct healthcare costs for constipation in Canada (CAD$)

Cost category	Cost ($ million)
Physician	73.1
Hospital	39.4
Serious hospitalisation	21.2
Drugs	80.0
Total costs	213.7

Data provided by the Canadian Institute for Health Information. Estimates on indirect costs do exist but are not well-understood and were excluded from this analysis.

## Results

Table 3 presents savings in constipation-related healthcare costs when fibre intakes are increased from 19.1 g/day for men and 15.6 g/day for women to levels that correspond to the IOM's levels of adequate intake (38 g/day for men and 25 g/day for women) (4). Given adequate intakes of dietary fibre, under the universal fortification scenario, our first set of analyses predicted total annual healthcare savings of CAD$31.9 million of avoided constipation costs. Under the optimistic scenario, a more moderate 50% success rate was assumed and resulted in predicted annual savings of CAD$16.0 million. With 15 and 5% success rates, the pessimistic and very pessimistic scenarios, respectively, predicted total healthcare savings of CAD$4.8 and CAD$1.6 million in avoided constipation costs annually.

**Table 3:** Potential constipation-related healthcare cost savings from dietary fibre intakes that correspond to the Institute of Medicine's adequate intake (CAD$ million)

	Scenario			
	
Direct cost category	Universal	Optimistic	Pessimistic	Very pessimistic
Physician	12.3	6.1	1.8	0.6
Hospital	5.5	2.7	0.8	0.3
Serious hospitalisation	3.0	1.5	0.4	0.1
Drugs	11.2	5.6	1.7	0.6
Total savings	31.9	16.0	4.8	1.6

Data represent constipation-related healthcare savings from increasing dietary fibre consumption from current levels (Table 1) to levels that correspond to the IOM's adequate intake cut-offs, estimated at 38 g/day for men and 25 g/day for women (4). The universal fortification represents the best-case scenario of potential constipation-related cost savings if the entire population were to consume adequate quantities of dietary fibre. The optimistic scenario is a medium- to short-term pragmatic estimate of the potential cost savings when 50% of the population increases intakes of dietary fibre. The pessimistic scenario is a practical short- to medium-term estimate of cost savings that could follow the increase in dietary fibre intakes among 15% of the population. The very pessimistic scenario represents the worst-case estimate when 5% of the population make the dietary change.

The second set of analyses indicated that each 1 g per day increase in intakes of dietary fibre could lead to total healthcare cost savings that range between CAD$0.1 and CAD$2.5 million annually given worst to best-case scenarios (Table 4).

**Table 4:** Potential constipation-related direct healthcare cost savings for each 1 g/day increase in dietary fibre intakes (CAD$ million)

	Scenario			
	
Direct cost category	Universal	Optimistic	Pessimistic	Very pessimistic
Physician	1.12	0.56	0.17	0.06
Hospital	0.39	0.20	0.06	0.02
Serious hospitalisation	0.21	0.11	0.03	0.01
Drugs	0.79	0.40	0.12	0.04
Total savings	2.51	1.26	0.38	0.13

The universal fortification represents the best-case scenario of potential cost savings in the entire population. The optimistic scenario is a medium- to short-term pragmatic estimate of the potential cost savings in 50% of the population. The pessimistic scenario is a practical short- to medium-term estimate of cost savings that could follow the 1 g/day increase in dietary fibre intakes among 15% of the population. The very pessimistic scenario represents the worst-case estimate when 5% of the population increase their dietary fibre intakes by 1 g/day.

As expected, in the third set of analyses, further total savings were evident with incremental increases in dietary fibre intakes (Table 5). In particular, when fibre intakes were increased from current levels to 20, 25, 30, and 35 g fibre/day (for men and women alike), annual CAD$0.4–8.0 million, CAD$1.0–20.6 million, CAD$1.7–33.2 million, and CAD$2.3–45.7 million savings in constipation-associated total healthcare costs were estimated, given the very pessimistic through universal assumptions, respectively.

**Table 5:** Total potential constipation-related direct healthcare cost savings with incremental increases in intakes of dietary fibre (CAD$ million)

	20 g/day	25 g/day	30 g/day	35 g/day
Universal	8.0	20.6	33.2	45.7
Optimistic	4.0	10.3	16.6	22.9
Pessimistic	1.2	3.1	5.0	6.9
Very pessimistic	0.4	1.0	1.7	2.3

Based on changes from current intakes of dietary fibre (Table 1), the universal fortification represents the best-case scenario of potential cost savings if the entire population was to increase daily fibre intake levels to 20, 25, 30, or 35 g/day (men and women alike). The optimistic scenario is a medium- to short-term pragmatic estimate of the potential cost savings when 50% of the population adopt the incremental increases in intakes of dietary fibre. The pessimistic scenario is a practical short- to medium-term estimate of cost savings that could follow the incremental increases in dietary fibre intakes among 15% of the population. The very pessimistic scenario represents the worst-case estimate when 5% of the population make the dietary changes.

## Discussion

With the use of data from established peer-reviewed studies and recent monetary estimates, the present economic valuation revealed potentially significant savings in constipation-related costs within the Canadian healthcare system that could follow higher daily intakes of dietary fibres. Given the ‘very pessimistic’ through ‘universal fortification’ scenarios, if between 5 and 100% of Canadian men and women consumed the adequate level of dietary fibre as established by the IOM (4), estimated CAD$1.6 to CAD$31.9 million, respectively, would be realised as total annual savings in healthcare costs associated with constipation. Under any scenario, these are non-trivial savings resulting from prevention of a highly prevalent disease. Rising healthcare costs are a growing concern, especially in Canada where associated expenditures consume more than half of all public budgets. Any opportunity to reduce these costs should be fully explored.

In spite of frequently-reported beneficial health implications of dietary fibres, little knowledge is available on the economic value of greater habitual or recommended fibre intakes. To date we are aware of only two studies pertaining to the economic benefits of increased dietary fibres as they relate to reduced rates of constipation-associated cost in the United States (19) and the European Union (20). Similar to our observations, those studies provided evidence of an association between increasing dietary fibre intakes and cost savings (base case) that exceed USD$12 billion annually in the United States, 127 million in the United Kingdom, €8.0/7.0 million in Ireland, and €121 million in Spain. In the United States (19), under a best-case scenario, the healthcare cost reductions were estimated at USD$83.9 billion. Further to these estimates, we here provide first evidence of potential cost savings of per 1 g and from moderate increases in fibre consumption.

In countries with publicly funded healthcare systems, such as Canada, the economic costs of non-communicable diseases place a considerable burden on increasingly strained healthcare budgets. By addressing unhealthy eating habits in the population it is possible to realise significant public health and economic benefits. In Canada, recent research has shown that over 30,000 deaths could be averted or delayed annually if a greater portion of the population complied with healthy dietary recommendations (22). Moreover, similar analyses to ours show that other healthy dietary behaviours have the potential to result in substantial economic savings (41–46). It is well known that the benefits of dietary fibre extend beyond that of digestive regularity. Increased consumption of dietary fibre is associated with reduced risk of severe lifestyle-related diseases (47, 48). Recent cost-of-illness analyses by our group have shown that modest increases in Canadians’ consumption of cereal fibre could also lead to substantial reductions in socioeconomic costs that are in turn linked to type 2 diabetes and cardiovascular disease (21). Thus, while the focus herein is on healthcare expenses associated with constipation, there are evidently broader and more impactful health-related economic benefits that stand from enhancing fibre intakes in Canada.

The present research underscores the importance of communicating the health benefits of dietary fibres to the general public and stakeholders alike. For instance, if the food industry is able to provide products that are acceptable and affordable by consumers, higher success rates and thus substantial savings could result. Furthermore, governments at all levels need to set clear guidelines as to the type of health claim(s) that could be made to ensure that a consistent and credible message is received by the consumer. Healthcare professionals can also play an important role in educating the public on the importance of fibre in the daily diet.

This work possesses a number of strengths. It is to our knowledge the first to examine the potential savings in healthcare costs attributed to higher intakes of dietary fibre and lower constipation rates among adults in Canada. The success rates, disease reduction, and adjusted healthcare cost data were possible through the most recent literature and national databases, while sensitivity analyses of varying scenarios enabled coverage of a wide range of assumptions for better predictions. A limitation of the present research is the lack of information relating to the indirect costs of constipation; although there is likely no significant mobility cost component, a reduction in short-term losses to productivity could present additional economic benefits. Also, a lack of information on consumer behaviour as it relates to the consumption of fibre requires fairly broad assumptions to be applied to the general population. Lastly, data on the prevalence of constipation in Canada are only available as of 2001, which is somewhat dated. It is, however, not likely that the Canadian population has experienced significant changes in constipation rates, although the prevalence may have changed. Also, in line with our assumptions, a recent review by Schmidt et al. (49) identified a constipation prevalence range of 2.6–26.9%.

## Conclusions

The present novel monetary estimates extend beyond the direct health benefits of dietary fibre into the realm of public healthcare budgets. Hence, healthy-eating behaviours that are consistent with the recommended levels of dietary fibre intake should be advocated as a practical approach for reducing costs associated with the management of constipation among adults in Canada.
